# Case of Pleuro-Pneumonitis

**Published:** 1845-07-01

**Authors:** E. R. Long

**Affiliations:** U. S. A. and the Editor


### Case of Pleuro-Pneumonitis.

Reported for the Buffalo Medical Journal, by Lieut. E. R. LONG, M. D., U. S. A. and the Editor.

Sullivan, Soldier, aged 22, of a robust constitution, after lying on the ground in a state of free perspiration and fatigue after violent exercise, was attacked, April 11th, with acute pain in the right side; tenderness on pressure between the ribs; respiration frequent and very painful; great thirst; tongue furred; pulse 105 and hard. Received into Hospital, under charge of Surgeon R. C. Wood, U. S Army; bleeding generally and topically, at once resorted to, with cathartics and diaphoretics.

12th, A. M. Pain Subsided, Pulse 90, Respiration 40, — P. M., Pain returned with great violence; suffocative respiration. Copious venesection and cupping repeated, with great relief. Physical signs—dulness up to the level of the nipple, anteriorly on the right side; above this sonorous *rale* anteriorly and posteriorly.* When lying on the left side inclining toward his face, the resonance of the lateral division of that side below axilla, good; on changing his position and lying on the back percussion over the regions just mentioned, yielded a flat sound. The act of moving, produces acute stitch; speaks with difficulty from the same cause. Diagnosis — Pleuritis with effusion.

14th. Entirely free from pain; pulse small; great prostration; was bled freely last evening for recurrence of severe pain. He has lost about 72 oz. of blood by venesection and cupping. Large blister over right anterior chest, yesterday. Physical signs; vesication prevented examination anteriorly;

* This may have been tubular respiration, [see subsequent history of the case] the two being liable to be confounded without care. If so the lung had already passed from the first to the second stage of Pneumonitis. Bronchial or tubular respiration, according to Laenec, may be present before the condensation is sufficient to produce flatness on percussion.—Ed.

posteriorly, dullness extends above lower margin of the middle third; above this, respiration is bronchial, with slight broncophony; on the left side, intensity of respiratory murmur increased.

15th. Very distressing cough, apparently dependent in a measure on meteorism, and relieved by the operation of an enema. At evening pulse 130. The physical signs render certain the existence of pneumonitis. Posteriorly there is dullness over the whole right side, with bronchial respiration down to the inferior third, and distinct broncophony; vesication prevents exploration anteriorly. From the general character of the vital and physical signs at the outset of the disease, it was regarded as pleuritis, and the characteristic sign of a complicating pneumonitis either overlooked, (crepitant rale) or it had ceased before the first examination.

16th, A. M.—Pulse 100; face flushed; cough less painful; *no expectoration has as yet appeared.* Bronchial respiration and broncophony over right side. Evening exacerbation, as, also, on previous days. Pulse 120; cough more painful.

17th—Aspect and general symptoms improved. Near the inferior angle of scapula, slight sub-crepitant rale.

18th—Much improved.

19th—Pulse 90; tongue covered with brown fur. *Still no expectoration.*

22d—Pulse 100; dullness and bronchial respiration continue; slight sub-crepitant rale in sub-mamary region.

23d—Cough distressing; sub-crepitant rale continues; Epistaxis last evening; pulse 80; feeble; tongue pale red, and nearly clean. Physical signs the same. Can rest comfortably only on back; turning on to left side excites distressing cough.

28th—Pulse 70; tongue clean. Respiration still accelerated. *No expectoration.*

29th—Bronchial character of respiration much diminished. Resonance on percussion improved, but still dull. He is dressed and permitted to walk out. Appetite good.

This was a case of Pleuro-Pneumonitis, a combination which is not now regarded as frequent, the pleuritis so often accompanying Pneumonitis, being considered to be a secondary complication in most instances. Acute Pleuritis here existed from the first, and from the severity of its symptoms masked the first stage of the Pneumonitis.

The efficacy of copious and repeated blood-letting was very manifest in this case. The patient was placed in imminent danger from the simultaneous attack of the disease upon the two structures, and the sudden interruption of the function of hematosis in the right chest. The relief from the successive bleedings was marked, and the prompt and bold use of this potent remedy probably saved the patient.

The rapidity of the convalescence as indicated by the general symptoms and the local signs is also an interesting feature, evincing the vigour of the patient's constitution.

The absence of expectoration is another striking circumstance. This, although rare, has been noticed by authors; Andral gives an account of a case of pneumonitis in which neither cough nor sputa were present.

The advantage of physical signs was very manifest. The symptoms would alone indicate very severe pleuritis. In the absence of the characteristic viscid, rusty sputa of pneumonitis, the latter could hardly have been diagnosticated without the aid of physical exploration.

The reporters of this case are indebted to the politeness of Dr. Wood for the opportunity of witnessing its progress under his judicious management, and for permission to report it for the Journal. Dr. Wood has, also, obligingly offered every facility to report interesting cases which may hereafter occur at the Hospital connected with the military post at this place.

*May 3d*, 1845.

